# Mechanical Properties of PEEK Post-Cores Compared to Other Post-Cores: A Systematic Review and Meta-Analysis

**DOI:** 10.1055/s-0045-1806952

**Published:** 2025-05-07

**Authors:** Binoy Mathews Nedumgottil, Muhammad Faheemuddin

**Affiliations:** 1 Department of Prosthodontics and Implantology, College of Dentistry, King Faisal University, Al Hassa, Saudi Arabia

**Keywords:** polyetheretherketone, systematic review, endodontics, endodontic post, post-cores

## Abstract

Recently, polyetherpolyetherketone (PEEK) has gained popularity as a dental biomaterial. However, there is a lack of consensus on its potential use as an endodontic post-core material. The aim of this review was to systematically critique and synthesize the evidence on PEEK-based post-cores in comparison to other materials. An electronic search was conducted on PubMed, Medline, Embase, ISI Web of Science, Ovid, Cochrane, and ClinicalTrials.Gov using relevant keywords. Seven *in vitro* studies were included in this review. Meta-analysis of fracture resistance was carried out on results reported in three studies. Overall, in most of the studies, PEEK post-cores performed similar to glass-fiber post-cores. The meta-analysis revealed no significant difference between the fracture strengths of PEEK and glass-fiber post-cores. However, in most studies, several sources of bias were identified. Within the limitations of this review, it may be concluded that mechanical and physical properties of PEEK posts are similar to those of glass-fiber post-cores. Nonetheless, long-term clinical studies are required to translate these conclusions into clinical practice.

## Introduction

For the restoration of teeth with inadequate tooth structure, post-and-core involves cementation of an endodontic post into an endodontically treated root of the tooth to retain the crown. 1 Several factors impact the survival of a crown retained by an endodontic post. These include length of the post relative to the root, remained tooth structure, and the ferrule available. 2 In addition to the aforementioned factors, the choice of material for endodontic posts is also crucial. Traditionally, metal posts (usually made of alloys such as cobalt-chromium or nickel-chromium [Ni-Cr]) have been used to construct post-and-core systems. 3 However, owing to the much higher elastic modulus of alloys compared to natural tooth, stresses may build up in the root, which may lead to vertical root fractures. 4 Additionally, the dark color of these alloys allows them to be visible over the gingival sulcus or even underneath the translucent ceramic crowns, compromising their esthetics and limiting their use in the anterior teeth. 5

To overcome the limitations of metallic posts, other materials have been considered for the construction of endodontic posts. These include fiber-reinforced glass posts, zirconia, and polymeric materials. 6 7 8 Another advantage of using these materials is their ability to be milled to endodontic posts chair-side via computer-aided design/computer-aided manufacturing (CAD/CAM) systems. 7 This not only shortens the treatment time and the number of appointments required but also allows construction of more accurate endodontic posts. 9 However, a recent systematic review of clinical trials suggests that the survival of glass-fiber posts is similar to those of alloy posts. 10 Studies comparing the survival of zirconia posts to metal posts are limited. 11

Polyetheretherketone (PEEK) is a thermoplastic polymer that has been used extensively to construct orthopaedic appliances such as prosthetic joints. 12 More recently, due to its excellent biocompatibility, esthetics, and mechanical properties, PEEK has been studied for its potential applications in fixed and removable prosthodontics, craniofacial constructive surgery, and dental implant therapy. 13 Like fiberglass and zirconia, PEEK can be milled in CAD/CAM systems. 14 Furthermore, the elastic modulus of PEEK is lower than natural tooth and bone. 15 Therefore, studies have shown that it distributes stresses more favorably compared to materials such as fiberglass and alloys. 16 Additionally, studies have also compared the mechanical properties and retention of PEEK posts to those of other materials. 17 However, due to lack of peer-reviewed systematic reviews, the overall consensus on the performance of PEEK posts relative to other materials is uncertain. Therefore, the purpose of this systematic review was to systematically critique and summarize the studies that have compared PEEK posts to those made of fiberglass, metal alloys, zirconia, and other materials.

## Methods

### Focused Question and Protocol Registration

Using the Participant, Intervention, Control, and Outcomes principal described in the Preferred Reporting Items for Systematic Review and Meta-Analyses statement, 18 a focused question was constructed. The focused question was: “Are the survival rates, mechanical properties, failure, and overall performance (outcomes), of teeth restored (participants) with PEEK-based post-and-core systems (intervention) better or worse when compared to non-PEEK-based control posts (e.g., fiberglass, zirconia, alloys)?” The PRISMA checklist in provided in Appendix A.

### Selection Criteria and Literature Search

Studies that compared PEEK-based post-and-core systems with any other material were included. The following outcomes were included: mechanical properties, survival, failure modes, and overall performance. All types of comparative studies (clinical, *in vitro* , *in vivo* , and *ex vivo* ) studies were included. However, studies conducted on animal teeth, case reports, noncomparative cohort or observational studies, systematic reviews, nonpeer-reviewed conference abstracts, opinions, and letters to the editors were excluded. There was no language restriction on the inclusion of studies. Any non-English studies were translated using Google Translator. Any studies that could not be translated were excluded during the full-text screening process. Any conflicts were solved by discussion. The following MeSH terms and Boolean characters were used: [((polyetheretherketone) OR (PEEK)) AND ((endodontic) OR (root canal) OR (crown))) AND ((post) or (core) or (post-and-core)) AND ((survival) OR (mechanical) OR (fracture) OR (retention) OR (adhesion) OR (survival) OR (failure) OR (success))]. The following databases were searched from their inception to November 1, 2023: PubMed, Medline, ISI Web of Science, Embase, Ovid, Cochrane, and ClinicalTrials.Gov. Gray literature search was conducted via assistance from the Library Services at King Faisal University and through Google Scholar. Additionally, a monthly update search was conducted until completion of this projection.

### Data Extraction

The data extraction was carried out by two reviewers independently into precalibrated Microsoft Excel forms. Briefly, data pertaining to the following categories was extracted from the studies: type of study, sample size and types, study groups, the type of adhesion systems used, and outcomes. Additionally, outcomes were summarized qualitatively. The general characteristics and qualitative description of the reported outcomes are provided in Table 1 . For qualitative analysis (meta-analysis), numerical values were extracted from similar study groups across studies and pooled.

**Table 1:** General characteristics and outcomes of the included studies

Study (author, year)	Type of study	Sample, type, and size ( *n* )	Study groups ( *n* )	Adhesion system	Outcomes assessed	Overall outcomes
Benli et al, 2020 19	*In vitro*	60 human maxillary incisors	PEEK ( *n* = 20) Glass fiber ( *n* = 20) Cast metal ( *n* = 20)	Self-etch resin	Pull-out test (tensile bond strength) Surface roughness Failure modes	GF posts had the highest SR and lowest TBS values; PEEK had the highest TBS and lowest SR values
Teixeira et al, 2020 20	*In vitro*	48 mandibular premolars	PEEK ( *n* = 12) Nanoceramic composite ( *n* = 12) Cast metal (NiCr) ( *n* = 12) Custom nanohybrid composite ( *n* = 12)	Self-etch resin	Fracture resistance	PEEK posts comparable with GF posts but significantly lower than metal posts ( *p* < 0.0001) Cast metal posts had a higher incidence of nonrepairable failures ( *p* < 0.001)
Özarslan et al, 2021 21	*In vitro*	120 maxillary incisors	PEEK ( *n* = 40) Zr ( *n* = 40) Glass fiber ( *n* = 40) Each group divided 3 subgroups: narrow posts (1.4 mm W; L: 7.5 and 10 mm; *n* = 10) and wide posts (1.6 mm drill; L: 7.5 and 10 mm; *n* = 10)	Dual-cure resin	Fracture resistance Failure analysis	PEEK post-cores showed sufficient fracture strength for the anterior region, similar to the other two post-core materials. PEEK post-cores showed more decementation and repairable fractures at lower forces, while restoration failure was mostly catastrophic for glass fiber and zirconia posts
Kole and Ergun, 2023 22	*In vitro*	256 maxillary central incisor	PEEK ( *n* = 64) PEKK ( *n* = 64) Zr ( *n* = 64) Glass fiber ( *n* = 64) Each divided in to 4 subgroups: (1) 10 mm L, 1.75 W ( *n* = 16) (2) 10 mm L, 1.5 mm W ( *n* = 16) (3) 7 mm L, 1.75 mm W ( *n* = 16) (4) 7 mm L, 1.5 mm W ( *n* = 16) All subgroups divided into cyclic loading (250,000 cycles, 50 N at 37°C)	Dual-cure resin cement	Push-test	Bond strength values of Zr had the highest values ( *p* < 0.05) without cyclic loading. PEEK (7 mm L and 1.75 mm W) had the weakest bond strength without cyclic loading. After cyclic loading, all posts had similar bond strengths
Pourkhalili and Maleki, 2022 23	*In vitro*	33 premolars	Fiberglass ( *n* = 11) Cast metal ( *n* = 11) PEEK ( *n* = 11)	Resin cement (curing not specified)	Fracture strength Failure analysis	Fracture resistance was significantly higher in the NiCr post than in the fiberglass and PEEK posts ( *p* < 0.001). More repairable fractures in PEEK posts
Rakotoaridina et al, 2023 24	*In vitro*	40 maxillary molars	PEEK/TiO _2_ ( *n* = 10) Ceramic/polymer ( *n* = 10) Glass fiber/epoxy ( *n* = 10) Glass fiber/bis-GMA ( *n* = 10)	Self-adhesive resin	Fracture strength Fracture analysis	PEEK/TiO _2_ post had lower compression fracture resistance compared to glass fiber-based posts ( *p* = 0.005) but higher than ceramic-based posts
Saisho et al, 2023 17	*In vitro*	80 premolars	PEEK ( *n* = 20) Composite resin ( *n* = 20) Polymer-ceramic ( *n* = 20) Glass fiber epoxy ( *n* = 20) Subgroups: Thermocycling (3,000 cycles, 55°C)	Dual-cure	Mechanical fatigue Pull-out bond strength Fracture analysis	All materials had statistically similar fatigue strengths ( *p* = 1.60). Glass fiber posts had significantly higher ( *p* < 0.05) bond strength than PEEK

Abbreviations: GF, glass fiber; GMA, glycidyl methacrylate; NiCr, nickel-chromium; PEEK, polyetherpolyetherketone; PEKK, polyetherketoneketone; SR, surface roughness; TBS, tensile bond strength; TiO _2_ , titanium oxide; Zr, zirconia.

### Meta-Analysis

**Fig. 1:**
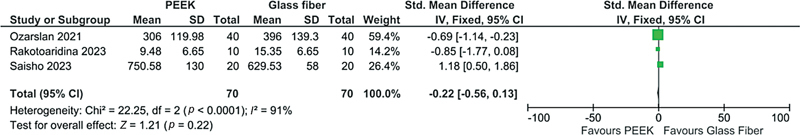
Results of the meta-analysis.

Using RevMan 5.4 software, 25 the pooled data from studies was analyzed. Briefly, standard mean differences (mean and standard deviations) from comparable test and control study groups were pooled using a random effects model. Statistical significance was set at 0.05 and the *I* ^2^ statistic was carried out to deduce the heterogeneity of included studies. Results of the meta-analysis are illustrated in Fig. 1 .

### Risk of Bias Assessment

As dictated by the type of studies included in this review, Quality Assessment Tool for In Vitro Studies (QUIN Tool) was used to assess the quality and risk of bias of studies included in this review. 26 Briefly, QUIN is a scale that assesses risk of bias in the following domains in *in vitro* studies: aims and objectives, sample size, explanation of sampling techniques, details of comparison group, explanation of methodology, operator details, randomization, method of outcome measurements, details of outcome assessors, blinding, statistics, and results.

## Results

### Literature Search

**Fig. 2:**
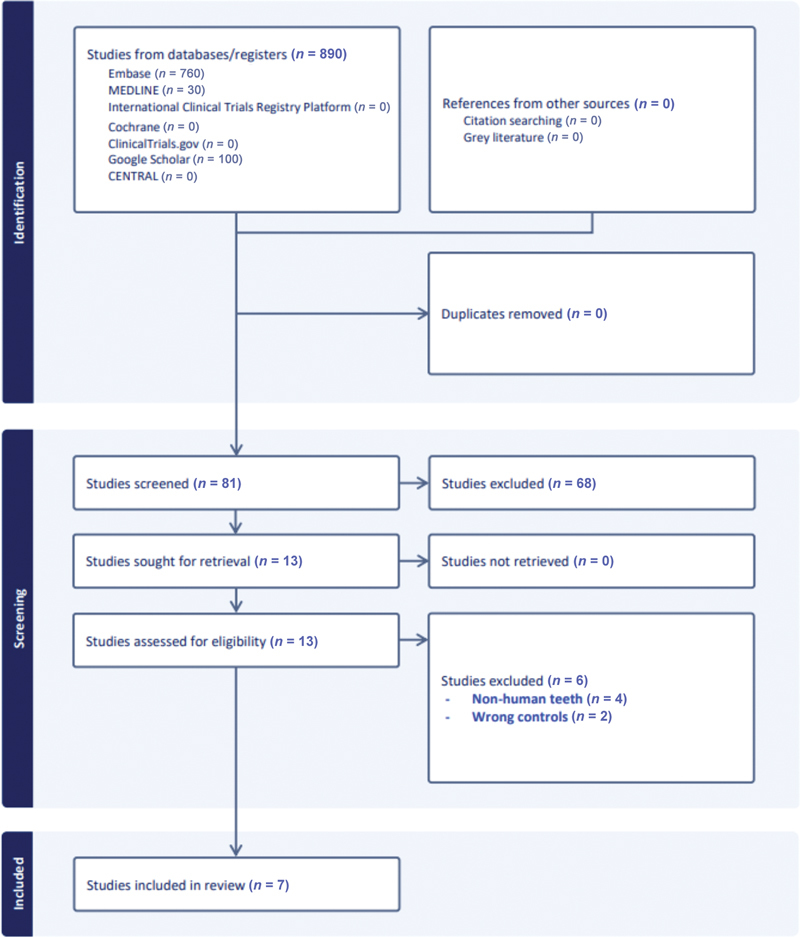
Preferred Reporting Items for Systematic Review and Meta-Analyses (PRISMA) flow diagram of the literature search conducted for this review.

Initial search resulted in 890 studies. Of these studies, 799 studies were excluded because they were irrelevant to our review and 10 duplicates were identified, leaving 81 studies for abstract screening. Of these 81 items, 13 items were selected for full-text screening. 17 19 20 21 22 23 24 27 28 29 30 31 32 Six studies were further excluded because of two reasons: (1) nonhuman teeth 27 29 30 32 and (2) wrong controls. 28 31 Thus, seven studies were ultimately included in this review. 17 19 20 21 22 23 24 Details of the literature search is illustrated in Fig. 2 .

### General Characteristics of Studies

All studies had an *in vitro* study design in which PEEK-based post-cores were manufactured and cemented in extracted human teeth. 17 19 20 21 22 23 24 Number of teeth restored with post-cores ranged from 33 to 256. 17 19 20 21 22 23 24 In the seven included studies, the following comparators were used: glass fiber 17 19 21 22 23 24 (in six studies), cast metal (in three studies 19 20 23 ), resin composites (in two studies 17 20 ), polyetherketoneketone (PEKK; in one study 22 ), polymer-ceramic composite (in two studies 17 24 ), zirconia (in one study 22 ), and PEEK-TiO _2_ composite (in one study 24 ). Self-etch or -adhesive resins were used in three studies for cementing post-cores, while self-cure resins were used in the other three studies. In one study, the type of resin system was not specified. 23 Majority of the studies focused on fracture strength and analysis, 17 19 21 23 24 three studies also focused on measuring the bond strength via pull-out or push-out testing. 17 19 22 Furthermore, one study also measured mechanical fatigue. 17 Thermocycling was carried out only in two studies. 17 22

### Overall Outcomes of Studies

In one study, PEEK posts had similar fracture strength to glass fiber posts but it was significantly lower than metal posts. 20 In another study, PEEK posts-cores had similar fracture strength to both, glass fiber and zirconia post-cores. 21 In another study, PEEK posts had significantly lower fracture resistance than Ni-Cr posts. 23 PEEK-TiO _2_ posts had a significantly lower compression fracture strength compared to glass fiber but higher than ceramic posts. 24 In one study, PEEK posts had similar fatigue strength compared to glass fiber and polymer-ceramic posts after thermocycling. 17 In the same study, however, glass fiber posts had significantly higher bond strengths than PEEK and polymer-ceramic. 17 Nevertheless, none of the PEEK post-cores suffered adhesive or cohesive fractures but rather went through core deformation instead. 17 Finally, in the study by Kole and Ergun, Zr post-cores had the highest bond strengths compared to both PEEK and PEKK post-cores. 22 The descriptive outcomes are presented in Table 1 while the numerical values of the fracture strength, adhesive strength, tensile strength, and the failure modes are provided in Table 2 .

**Table 2:** A summary of the outcomes reported in the studies included in this review

Study (author, year)	Fracture resistance (Newton or MPa), SD	Tensile bond strength (µm)	Surface roughness (Ra, µm)	Failure modes (%)
Benli et al, 2020 19	NR	PEEK: 14.33 ± 0.58 ( *p* < 0.001) Cast metal: 12.79 ± 0.39 Glass fiber: 10.05 ± 0.53	PEEK: 1.37 ± 0.11 ( *p* < 0.001) Cast metal: 2.52 ± 0.25 Glass fiber: 2.93 ± 0.18	Adhesive PEEK: 80%, cast metal: 60%, glass fiber: 65% Cohesive PEEK: 0%, cast metal: 10%; glass fiber: 0% Mixed PEEK: 20%, cast metal: 30%, glass fiber: 35% *p* = 0.243
Teixeira et al, 2020 20	PEEK: 379.4 ± 119.98 N Nanoceramic composite: 506.4 MPa ± 138 N Cast metal (NiCr): 939.6 ± 146.5 N ( *p* < 0.0001) Custom nanohybrid composite: 449.6 ± 66.5 N	NA	NA	NA
Özarslan et al, 2021 21	PEEK: 306 ± 74.0 to 371 ± 94.0 N Glass fiber: 396 ± 139.3 to 514.2 ± 136.3 N Zirconia: 289.5 ± 70.5 to 450.4 ± 109.5 N	NR	NR	NR
Kole and Ergun, 2023 22	NR	Maximum values No cyclic loading: PEEK: 5.44 ± 1.85 Glass fiber: 8.35 ± 3.02 Zirconia: 9.74 ± 6.20 PEKK: 6.95 ± 2.13 After cyclic loading: PEEK: 3.37 ± 1.29 Glass fiber: 5.51 ± 2.37 Zirconia: 6.25 ± 4.03 PEKK: 4.22 ± 1.46	NR	NR
Pourkhalili and Maleki, 2022 23	PEEK: 811.30 N NiCr: 1248.35 N ( *p* < 0.001) Fiberglass: 668.25 N SD values not available	NR	NR	NR
Rakotoaridina et al, 2023 24	PEEK/TiO _2_ : 9.48 ± 6.65 MPa Ceramic/polymer: 15.88 ± 4.37 MPa Glass fiber/epoxy: 15.35 ± 6.65 MPa Glass fiber/bis-GMA: 6.05 ± 4.14 MPa	NR	NR	Dental: PEEK/TiO _2_ : 80% Ceramic/polymer: 60% Glass fiber/epoxy: 70% Glass fiber/bis-GMA: 0% Material: PEEK/TiO _2_ : 10% Ceramic/polymer: 20% Glass fiber/epoxy: 0% Glass fiber/bis-GMA: 60% Mixed: PEEK/TiO _2_ : 10% Ceramic/polymer: 20% Glass fiber/epoxy: 30% Glass fiber/bis-GMA: 40%
Saisho et al, 2023 17	PEEK: 750.58 ± 130 MPa Composite resin: 595.74 ± 113 MPa Polymer-ceramic: 664.25 ± 117 MPa Glass fiber epoxy: 629.53 ± 58 MPa	PEEK: 1.27 Composite resin: 1.86 Polymer-ceramic: 1.67 Glass fiber epoxy: 2.11 (SD values could not be extracted)	NR	Types of failure: PEEK: Type 0 (100%) GF: Type 1: 80%; Type 2: 20% Polymer ceramic: Type 1: 40%; Type 3: 40%; Type 4: 20% Composite resin: Type 1: 50%; Type 2: 50%

Abbreviations: GMA, glycidyl methacrylate; NiCr, nickel-chromium; NR, not reported; PEEK, polyetherpolyetherketone; PEKK, polyetherketoneketone; SD, standard deviation; TiO _2_ , titanium oxide; Zr, zirconia.

### Result of the Meta-Analysis

Results of the fracture strength analysis from only three studies could be pooled. 17 21 24 The forest plot for the pooled results is presented in Fig. 1 . The results had a very high heterogeneity ( *I* ^2^ = 91%). Furthermore, there was no statistically significant difference between PEEK and glass fiber posts as far as fracture strengths are concerned. Meta-analyses of tensile bonding strength, failure modes, and surface roughness could not be conducted due to the lack of comparable outcomes.

### Result of the Risk of Bias Assessment

Six included studies were estimated to have a high level of bias, 17 19 20 21 22 24 and one was graded as having a low level of bias. 23 The details of the risk of bias assessment are presented in Table 3 .

**Table 3:** Results of the risk of bias assessment

Study (author, year)	Domain											
	Aims/Objectives	Sampling technique	Details of comparison groups	Methods	Operator details	Randomization details	Measurements	Outcome assessors	Blinding	Statistics	Results	Overall bias
Benli et al, 2020 19	Yes	Yes	Yes	Yes	No	No	Yes	No	No	Yes	Yes	High
Teixeira et al, 2020 20	Yes	No	Yes	Yes	No	No	Yes	No	No	Yes	Yes	High
Özarslan et al, 2021 21	Yes	No	Yes	Yes	No	No	Yes	No	No	Yes	Yes	High
Kole and Ergun, 2023 22	Yes	Yes	Yes	Yes	No	No	Yes	No	No	Yes	Yes	High
Pourkhalili and Maleki, 2022 23	Yes	Yes	Yes	Yes	Yes	Yes	Yes	No	No	Yes	Yes	Low
Rakotoaridina et al, 2023 24	Yes	No	Yes	Yes	No	Yes	Yes	No	No	Yes	Yes	High
Saisho et al, 2023 17	Yes	Yes	Yes	Yes	No	No	Yes	No	No	Yes	Yes	High

## Discussion

The mismatch in elastic modulus between natural tooth and significantly more rigid materials, such as cast alloys, is a key factor contributing to the fracture of teeth restored with post-core-retained crowns. 33 Finite element analysis of cast post-cores and glass fiber posts have revealed that there is a much higher buildup of stress in teeth restored with alloy post-cores because alloys possess a significantly higher modulus of elasticity than glass fiber posts, which have a modulus much closer to that of natural dentine. 33 Similarly, a comparison of cast post-cores and composite post-cores reveals that alloy post-cores lead to a higher of cervical fractures compared to composite post-cores. 34 Benli et al have observed that PEEK post-cores have a higher number of adhesive fractures compared to cast post-cores and glass fibers. 19 However, this difference was not statistically significant. Notably, in one study, all PEEK post-cores exhibited deformation rather than fractures when compared to glass fiber, polymer ceramic, and composite resin. 17 We hypothesize that the significantly less elastic modulus of PEEK would be an advantage when a post-core material is used, leading to a lesser number of unrepairable and catastrophic fractures. Our meta-analysis of PEEK and fiberglass suggests that there is no significant difference between the two materials in terms of fracture resistance. 17 21 24 Nonetheless, the extreme high heterogeneity of the outcomes impeded us from drawing any meaningful conclusions. The heterogeneity is most likely because of the differences in the fracture analysis and differences in the procedures such as cementation and preadhesive procedures.

*In vitro* studies on extracted teeth have limitations in directly translating to clinical observations for several reasons. First, the removal of a tooth from its natural environment disrupts the complex interactions between the tooth and the surrounding oral tissues, including the periodontal ligament, alveolar bone, and gingival tissues. 35 Moreover, tooth sterilization procedures required prior to *in vitro* experiments further deteriorates the tooth material. 35 Furthermore, *in vitro* studies on extracted teeth often involve artificial conditions and controlled environments that do not fully simulate the dynamic and variable nature of the oral cavity, where factors like saliva, oral microbiota, and mechanical forces are at play. Consequently, findings from *in vitro* studies on extracted teeth may not accurately reflect the complexities of clinical scenarios, including the effects of systemic factors, patient variability, and long-term responses. For a more comprehensive understanding and reliable clinical translation, *in vivo* studies involving intact oral environments are essential. A significant limitation of this review is the absence of clinical studies, as no comparative clinical research involving PEEK and other materials was available for inclusion. Therefore, it is imperative that clinical studies be carried out in which the performance and survival PEEK post-cores are compared to those of other materials such as glass fibers and alloys.

Because of the differences in the macro- and microscopic features of animal and human teeth, we excluded studies conducted on animal teeth for qualitative and quantitative analysis, but it is still worthwhile to discuss their outcomes in this review. Lima et al have observed that if ferrule is provided in the restoration design, there is no difference between the biomechanics of PEEK and glass fiber posts. 29 In another study on animal teeth, PEEK and glass fiber posts performed similarly on flared roots. 27 This further validates our hypothesis that biomechanics of PEEK posts are similar to those of glass fiber posts. Nonetheless, an interesting study would be to compare the impact of differing ferrule design on PEEK and non-PEEK posts. Our meta-analysis revealed a high proportion of heterogeneity in the outcomes. We hypothesize that it is because of the variability within the fracture testing protocol. Specifically, the rate of stress application varied between the studies (0.5–2.00 mm/min), 17 21 24 which could also account in the significant range of the fracture resistance (379.4 and 750.58 MPa). 17 21 Furthermore, none of these studies studied the impact of using different post materials on cemented crowns. Future studies that involve cemented crowns over PEEK and non-PEEK posts are suggested.

Results from this review suggest that PEEK post-cores are similar to glass fiber in terms of physical and mechanical properties. However, there are limitations that should be accounted for. First, we included only three studies in the meta-analysis with a high degree of heterogeneity, which makes it difficult to reach a consensus with a certainty. Second, we did not find any clinical comparative studies that could be included in this review, making clinical applicability of these outcomes debatable. Similarly, although PEEK is a promising material for removable prosthodontics, due to the lack of long-term clinical studies, its long-term survival is debatable. 36

## Conclusion

Within the limitations of this review and included studies (for example, small sample sizes and lack of clinical data), it may be concluded that mechanical and physical properties of PEEK posts are similar to those of glass fiber post-cores. Nonetheless, long-term clinical studies with larger sample sizes and human subjects are required to translate these conclusions into clinical practice.

**Appendix A:** PRISMA 2020 Checklist

Section	Item	Page Number
Title	Title	1
Abstract	Structured Summary	1
Introduction	Rationale	2
	Objectives	3
Methods	Eligibility Criteria	4
	Information Sources	4
	Search Strategy	4
	Selection Process	4
	Data Collection Process	5
	Data Items	5
	Study Risk of Bias Assessment	6
	Effect Measures	6
	Synthesis Methods	6
	Reporting Bias Assessment	NA
	Certainty Assessment	NA
Results	Study Selection	7
	Study Characteristics	7
	Risk of Bias in Studies	8
	Results of Individual Studies	8
	Synthesis of Results	9
	Heterogeneity	9
	Reporting Bias Assessment	NA
	Certainty of Evidence	NA
Discussion	Summary of Evidence	10
	Limitations	10
	Conclusions	11
Other Information	Registration and Protocol	NA
	Support	12
	Competing Interests	12
	Availability of Data, Code, and Other Materials	NA
